# Analyzing the Coevolution of Mobile Application Diffusion and Social Network: A Multi-Agent Model

**DOI:** 10.3390/e23050521

**Published:** 2021-04-24

**Authors:** Zhenyu Zhang, Huirong Zhang, Lixin Zhou, Yanfeng Li

**Affiliations:** 1 School of Automation, Nanjing University of Science and Technology, Nanjing 210094, China; zhangzhenyu@njust.edu.cn; 2 School of Labor Relationship, Shandong Management University, Jinan 250357, China; 14438120140358@sdmu.edu.cn; 3 Business School, University of Shanghai for Science and Technology, Shanghai 200093, China; 4 School of Economics and Management, Tongji University, Shanghai 200092, China; 1631023@tongji.edu.cn

**Keywords:** multi-agent, innovation diffusion, social network, mobile application, coevolution

## Abstract

The successful diffusion of mobile applications in user groups can establish a good image for enterprises, gain a good reputation, fight for market share, and create commercial profits. Thus, it is of great significance for the successful diffusion of mobile applications to study mobile application diffusion and social network coevolution. Firstly, combined with a social network’s dynamic change characteristics in real life, a mobile application users’ social network evolution mechanism was designed. Then, a multi-agent model of the coevolution of a social network and mobile application innovation diffusion was constructed. Finally, the impact of mobile applications’ value perception revenue, use cost, marketing promotion investment, and the number of seed users on the coevolution of social network and mobile application diffusion were analyzed. The results show that factors such as the network structure, the perceived value income, the cost of use, the marketing promotion investment, and the number of seed users have an important impact on mobile application diffusion.

## 1. Introduction

With the rapid development of mobile Internet technology, China’s mobile 4G network construction is in full swing. The 5G network is gradually being put into use, and Internet users’ access to information and product services through mobile devices has become increasingly convenient and efficient. Driven by the scale of mobile Internet users and the mobile Internet industry’s vigorous development, many mobile applications emerge as the times require. A mobile application is application software installed on smartphones or other mobile devices and a product or service innovation based on Internet technology. The successful diffusion of mobile applications in user groups can establish a good image for enterprises, form word-of-mouth, fight for market share, and create commercial profits. Simultaneously, the innovation of mobile applications also requires enterprises to invest a lot of funds and resources. The failure of mobile application diffusion in the market may bring huge economic losses to enterprises. Therefore, people expect to help Internet enterprises avoid innovation risks, improve the diffusion effect, and create more profit value by studying the spread of mobile applications in the market. As innovations based on mobile Internet technology, the spread and diffusion of mobile applications in user groups have attracted more and more scholars’ attention and interest. Mobile application innovation diffusion has become a hot issue in innovation diffusion research [1].

In real society, the social relationship between users is not static or fixed. There is an interaction between innovation diffusion and the network structure, also known as coevolution [2]. Social networks influence innovation diffusion, and whether individuals adopt innovation also affects the characteristics of the social networks [3]. Exploring innovation diffusion under symbiotic evolution is of considerable significance to understanding the essence of the internal diffusion mechanisms. Most current research on innovation diffusion often ignores network structure changes and the dynamic coupling between innovation diffusion and network structures [4]. Examining innovation diffusion under symbiotic evolution from the active network’s perspective is the key to exploring the essence of innovation diffusion and the direction to be further studied. Some studies have shown that the introduction of a network’s dynamic characteristics reasonably explains many unexplained phenomena under the static system [5]. Therefore, considering the dynamic changes of the network meets the needs of theoretical research on innovation diffusion from the complex system’s perspective and fits better with real situations of real society. The following is a detailed description of mobile application diffusion.

In mobile application diffusion, the user relationship’s social network structure is not a continuous static structure. Users often choose different objects to establish, or they disconnect the connection according to their decision-making utility and situation, resulting in a change of the overall social network structure. Social network diffusion and mobile application innovation diffusion influence each other. Specifically, users who adopt the same decision-making strategy are more likely to connect and become friends, presenting a convergence phenomenon [6]. Simultaneously, users may also lack communication and interaction for a long time, ultimately leading to the indifference and estrangement of social connection between them. For example, if a user loves a game application, they will probably become friends with another user who also loves the mobile application. However, suppose the user reduces communication with the neighboring users for some reason and does not interact for a long time. In that case, the relationship between the users may be indifferent, and the social connection between them may be challenging to maintain. This shows that the decision-making and interaction among users will, in turn, affect their social network structure, and the change in the social network structure will further affect the decisions of users and ultimately affect the results of innovation diffusion of mobile applications. So, will the complexity of a social network structure affect the distribution of mobile application innovation? Under the evolution of a social network, what are the characteristics of user group decision development? What is the relationship between them? What are the roles of various internal and external factors in the process of collaborative evolution? All these problems need us to explore the coevolution of social networks and mobile application innovation diffusion.

Zhou et al. [7] constructed a simulation model based on the static social network environment and compared mobile application diffusion results under different social network structures and network effects. However, the dynamic changes in network structures were not considered. Therefore, we considered the dynamic changes in social networks in the process of innovation diffusion, mainly studying the laws and the relationship between social networks and innovation diffusion, and the roles of various internal and external factors in the process of coevolution to gain further insight into the internal mechanisms and characteristics of mobile application innovation diffusion. Firstly, combined with the social network’s complex and changeable characteristics in real life, a social network’s evolution mechanism was designed, including old connection disconnection and new connection generation. A multi-agent coevolution model of social network and mobile application diffusion considering the local network effect was constructed. We present a discussion of the coevolution characteristics and the interaction between social network structures and user group decision-making evolution. Finally, various internal and external factors in the coevolution of social networks and innovation diffusion were analyzed.

The contributions of this paper are as follows. Firstly, a multi-agent model considering the dynamic changes in network structures was developed to simulate mobile application diffusion. Secondly, our findings show that factors such as the network structure, perceived value income, cost of use, marketing promotion investment, and the number of seed users have an important impact on mobile application diffusion. This provides firms with promotion strategies and suggestions for mobile application diffusion.

The rest of this paper is organized as follows. First, we introduce the literature on innovation diffusion, the application of a multi-agent model in innovation diffusion, and mobile application diffusion in Section 2. Section 3 provides the conceptual design of the developed multi-agent simulation model. The setting and the steps of the multi-agent-based mobile application diffusion model are explained in Section 4. The results of the simulation experiments are presented and analyzed in Section 5. Finally, some suggestions for marketing decision-making and management policy of enterprises are concluded in Section 6.

## 2. Literature Review

### 2.1. Innovation Diffusion

The concept of diffusion originates from physics and describes a mass migration phenomenon. The definition of innovation diffusion by Rogers has been widely recognized [8,9]. He thinks that innovation diffusion refers to how innovation spreads among social system members, through specific channels and over time. Innovation includes various forms, such as new technologies, new products, new ideas, and new systems.

The innovation diffusion model has been divided into two categories: the macro-model and the micro-model. In terms of the macro-model, Bass [10] proposed the Bass diffusion model in 1969, which laid the foundation for quantitative research on innovation diffusion, and regarded the innovation diffusion model as the key to innovation diffusion theoretical research reaching milestone significance. Since then, many scholars have adjusted and relaxed the original model assumptions based on the Bass model to expand its application scope. Kalish [11] relaxed the Bass model’s assumption that market potential remains unchanged in the whole diffusion, taking the attributes of new products as internal factors and market characteristics as functions of external factors, and taking into account possible changes in consumers’ attitudes and expectations of products. Easingwood [12] paid attention to the influence of word-of-mouth communication on consumers’ decision-making and considered this feature. By introducing the model’s construction, the model’s influence coefficient was adjusted through the dynamic influence effect, and the degree of rationalization was improved. Considering the diversity of non-adopters’ sensitivity to fashion, Tashiro [13] proposed a new diffusion model to fit iPod and iPhone sales data and achieved good results. The above models take the social system as the research object, explain the dynamic mechanism of innovation diffusion with a simple idea and a highly operational method, analyze the factors influencing innovation diffusion through a mathematical model, and explore innovation diffusion laws [14].

However, the research conducted from a macro perspective only reflects the overall diffusion speed and scale situation. It does not have insight into the micro process of inter-individual diffusion in innovation diffusion, and therefore, has certain limitations [15]. With increasing individual differences among users, user groups’ heterogeneity (such as price sensitivity or risk preference) has become a factor that cannot be ignored. This macro perspective can no longer truly reflect the specific situation of innovation diffusion. More and more scholars have begun to use the microsimulation model to study innovation diffusion from individual social members’ perspectives.

From the perspective of micro individuals, the essence of innovation diffusion should be user decision-making. The main idea of micro model research is to obtain the macro level’s innovation diffusion curve by accumulating each individual’s decision-making situation. The maturity of computer technology provides a good guarantee for this research method. Microsimulation models are used in innovation diffusion-related research, including the protocell diffusion model, dynamics model, threshold model, and multi-agent simulation model. Cadavid and Cardona [16] explored individual preferences and social network neighbors’ influence on consumers’ decision-making, indicating that the social network structure and consumers’ characteristics affected marketing strategies. Song [17] used a multi-agent simulation method to simulate two types of companies selling products and services to users with different pricing strategies, and studied the dynamic market changes under the two pricing strategies. It was found that the effect of subscription license pricing was significantly affected by the entry time of new products and technical advantages. Therefore, the research on innovation diffusion has evolved from the macro diffusion model to the complex system model based on the micro individual perspective. The micro model is more and more used in innovation diffusion research because it can reveal macro phenomena and micromechanisms. Therefore, this paper also follows the idea of studying mobile application diffusion in user groups from the micro-level. By accumulating users’ individual decision-making results, we combined the micro behavior of user decision-making in complex systems with the innovation diffusion of mobile applications to better explore mobile application innovation diffusion’s internal mechanism and laws.

### 2.2. Applications of Multi-Agent Model in Innovation Diffusion

The multi-agent simulation method can reflect the dynamic process of micro behavior evolving into the macro phenomenon in complex systems by simulating the behavior and interaction of multiple agents in the system, consistent with the micro adoption and macro diffusion of innovation in the research of innovation diffusion. In recent years, agent-based modeling and the simulation method have been adopted by many scholars to study innovation diffusion. Stummer et al. [18] constructed an agent model from the two dimensions of time and space to simulate repeated purchase decisions in the multi-product competitive market. Amini [19] applied the agent simulation method to study production and sales policies’ influence on new products’ diffusion revenue. Kiesling et al. [20] concluded the strengths and limitations of agent-based modeling in innovation diffusion, discussed new insights provided by agent-based models, and outlined future research prospects. Wang et al. [21] explored technical innovation and its diffusion process in China by constructing an agent-based innovation diffusion model. The results showed that most innovative firms were located in the east of China, and preferential policies can accelerate innovation diffusion to less developed regions. Jiang [22] discussed some essential elements of diffusion, such as diffusion actors, diffusion media, and diffusion contents, and concluded some widely used diffusion models. Zhang et al. [23] used the maximum likelihood estimation framework to calibrate agent-based models for innovation diffusion, and the new model had advantages over the classical agent-based models. It has been proved that multi-agent simulation is an effective method to study innovation diffusion.

However, there are still some limitations in the current research, which needs more attention to be paid to the innovation diffusion of mobile applications. The existing simulation models have oversimplified the attribute characteristics of agents, especially decision-making and interaction. Most of them consider word-of-mouth effect and preference differences and do not involve complex agent interactions, such as strategy learning and decision imitation among individuals. There is a big difference between simulation models and the complex decision-making process and users’ mechanisms in real life. It needs to be further explored from an interdisciplinary perspective. Therefore, when using multi-agent simulation to study mobile application innovation diffusion, user agents’ attribute characteristics, behavior methods, and interaction rules were set to better reflect the complex decision-making process and mobile application users’ mechanisms in real society.

### 2.3. Mobile Application Diffusion

At present, the related research on innovation diffusion of mobile applications mainly focuses on the analysis of factors influencing the use or adoption behavior and intention of mobile applications combined with other theories, such as innovation diffusion. Based on innovation diffusion theory, information overload theory, and cluster behavior theory, Liu et al. [24] studied how personal views on innovation characteristics and social factors affect the uncertainty of mobile applications’ adoption. This can play an important role in reducing the uncertainty of adoption decision-making. Min et al. [25] studied the mobile application Uber’s adoption through innovation diffusion theory and a technology acceptance model. The results showed that the relative advantages, compatibility, and observability were obvious. Lu et al. [26] explored the factors that affect mobile applications’ adoption by tourists in rural tourism attractions. They found that the perceived usefulness, perceived ease of use, and compatibility were important prerequisites for users to adopt mobile applications. At the same time, self-efficacy indirectly affects users’ adoption intentions by adjusting the expected results. Chiu et al. [27] constructed the Technology–Organization–Environment (TOE) framework to analyze the critical factors of enterprises’ adoption of broadband mobile applications. Yi et al. [28] found that observability, complexity, and relative advantages were not the critical factors affecting health-related mobile applications’ adoption. The above research is the qualitative or empirical analysis of the factors that affect mobile applications’ adoption.

However, most of the research methods are static, and the influencing factors and their relationships have been analyzed from the social phenomena and existing data. Mobile application diffusion is a dynamic process in which users interact and decide to adopt mobile applications. At different stages, users’ psychological activities and individual behaviors are in different states. Finally, micro user decisions emerge as macro diffusion results with time evolution, which is a social phenomenon with time-varying delays. The above research ignores the social network environment in which mobile application innovation diffusion occurs and does not consider factors such as user heterogeneity, complex decision-making interaction between users, user relationship structure, or mobile application network externality. Hence, the exploration of the process and laws of mobile application innovation diffusion is very limited. Therefore, we focused on the process of mobile application innovation diffusion in the social network environment and considered more complex diffusion cases to better reveal the internal mechanisms and characteristics of mobile application innovation diffusion.

## 3. Conceptual Design of the Multi-Agent Simulation Model

The developed simulation model considers the complex and changeable characteristics of a social network structure. We introduced the network evolution mechanism in which the old connection is disconnected and reconnected, and a new connection is generated. The model’s design mainly includes the mobile application hypothesis, user decision-making, interaction process design, social network hypothesis, and social network evolution rule design. The focus of the developed model is the evolution of social networks. At the micro-level, the mechanism of the user’s adoption or rejection of mobile applications is the same as that of Zhou et al. [7], including the mobile application hypothesis, the conceptual model for individual decisions by a mobile application user, and the design of the mobile application user decision and interaction process. Next, we introduce the hypothesis of social network evolution and the design of social network evolution rules.

### 3.1. Social Network Evolution Hypothesis

In the real world, social networks are not static and stable; on the contrary, their topological structure is often in the process of dynamic change. At different times, there may be different relationships among nodes in a network. Inevitably, some nodes will change the existing connection mode, choose to disconnect from the current neighbor node, or establish a new connection relationship with other nodes. In the socio-economic context, two strangers become friends, the original trading partners stop business contact, and the cooperation between employees is interrupted. This behavior of individuals changing their connection status may also occur with multiple agents in the whole network, causing drastic changes in the overall network topology at a certain time. Although a shift in individual connection is not a high-frequency event for most of the complex networks in the real world, it will lead to a change in the social network structure after a considerable accumulation period.

The changes in social networks and social networks in real life are extremely complex. We make the following assumptions about the social network environment of mobile application innovation diffusion.

(1) The scope of the social network composed of mobile application users will not be expanded. New users will not be considered to enter the mobile application market. The number of users participating in the innovation and diffusion of mobile applications is certain, ensuring that the number of nodes of a social network will not change.

(2) Although there is some randomness in the change of a social network structure, it is not completely random. That is to say, the generation, disconnection, and reconnection of social connections among users follow some mechanisms and rules, and are related to the communication, interaction, and decision-making among users.

(3) There is no difference in the strength and type of social relations among users. That is, the neighborhood relationship is homogeneous, and the social network is still unauthorized.

### 3.2. Evolution Rule Design of Social Network

In mobile application innovation diffusion, users often choose different objects to establish, or disconnect relationships according to their value utility and decision-making situation. With the accumulation of time, the overall social network structure of users has changed. In this paper, the network evolution rules include the generation rules of new connections and the disconnection and reconnection rules of existing connections.

(1) The rules for generating new connections in the networks are as follows:

The conditions of connection formation will not be treated in the same way in different research fields applied to different subjects. For example, the users’ income value from a connection will be generally considered in a cooperative game. When the users’ sum income value can be increased due to a connection’s establishment, the connection between the users may be generated [29]. In mobile application diffusion, the users often establish social relations with different objects according to the value utility and decision-making situation [30,31,32]. When users and other users choose the same decision-making strategy, they are likely to communicate and interact [33,34]. For example, if a user considers adopting a game application, they are likely to interact and establish contact with users who also use the mobile application. The change of the user’s social relationship caused by the same decision-making state is described. When the user and other users who do not have contact originally adopt the same decision-making strategy in multiple time steps and reach a certain critical time step, social contact may occur between the two users. The connection between user agents will be generated in a probabilistic way, and the probability is represented by the parameter *Newconnect*.

(2) The disconnection and reconnection rules of existing connections in the network are as follows:

Users have social learning behavior in the evolutionary learning stage of user decision-making and interaction. Through an intelligent learning method, users can learn and imitate the highest value utility of mobile applications among the neighboring users and take the decision-making strategy of the imitating object at the same time as their next decision-making moment. On the one hand, as individual users connect through social networks, they imitate their neighbors’ decision-making through social learning behavior. On the other hand, they may become the object of decision-making learning and imitation of their neighbors [35,36,37]. When the users and the neighboring users have not learned to imitate for a long time, users will think that the neighbor user is not suitable for social learning, and will gradually reduce their communication and interaction, forming a perception of indifference and separation between them. When the neighbor user also generates this perception, users can no longer maintain this social connection [38,39]. Therefore, there is no one-way or two-way decision-making learning imitation between two neighboring agents in a certain time range. It is considered that when the relationship between users is indifferent and alienated, the connection between agents is disconnected by way of probability triggering, and the parameter *Disconnect* expresses the disconnection probability. The application scope of the neighbor agent’s disconnection condition is limited to the neighbor user in the network’s initial state. Still, the new neighbor relationship obtained by the new connection generation rule is considered. Moreover, the disconnection of the connection is not permanent. When the agent’s decision-making is the same as that of the original neighbor at a certain time, the agents will likely recover the previous neighbor contact due to reaching a consensus on the decision, and will reconnect the previously disconnected edge with a certain probability. The new connection generation rule’s differences are that the historical neighbor does not need to meet multiple time decision-making conditions because of social relations. Still, it only needs to reach a decision consensus at a certain time to carry out the reconnection. The probability of reconnection is expressed by the parameter *Reconnect*.

Besides each time step’s social learning behavior, the individual agent performs a new connection generation and an existing connection disconnection and reconnection judgment operation according to the above network evolution rules in each time step. Suppose the judgment conditions do not meet the network evolution conditions or fail to trigger the connection generation or disconnection in the form of probability. In that case, the individual agent cannot operate again in the current time step to ensure that the interaction response of the user’s intermittent connection is sequential. The network structure is a dynamic evolution.

## 4. A Simulation Model with Multi-Agents

The multi-agent model of the coevolution of a social network and mobile application innovation diffusion was constructed in Anylogic 8. The main context includes four parts: the setting of the main model and class, the realization of user decision-making and interaction process, the realization of network structure evolution, and the setting and verification of the model parameters.

### 4.1. The Setting of the Main Model and Category

**Figure 1:**
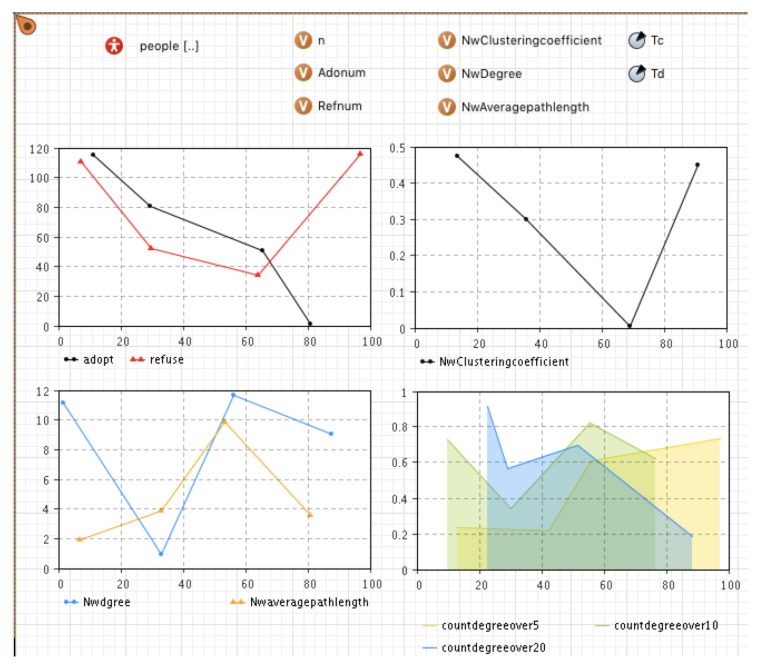
The main multi-agent model.

**Figure 2:**
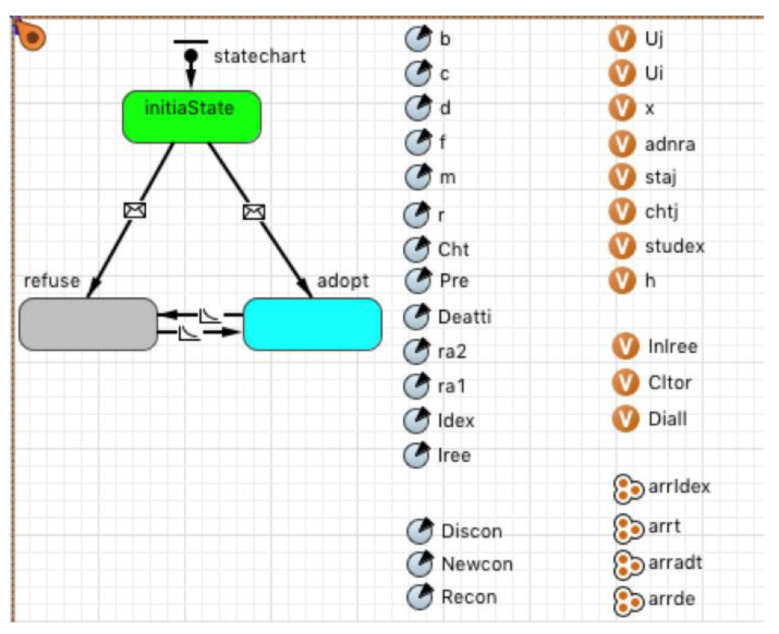
The Person category.

According to the conceptual simulation model mentioned above, a multi-agent model of mobile application diffusion considering the evolution of the social network was established in Anylogic 8 and is shown in Figure 1 and Figure 2. Figure 1 shows the developed multi-agent model. The “People” category refers to the collection of mobile application user agents, and each user is an agent. The other three charts show the changes in the above three statistical indicators of the network structure over time in the diffusion process. The network degree distribution chart adopts the broken line accumulation chart, showing the change of the number of nodes whose network medium value is more than 5, 10, and 20.

Figure 2 shows the “Person” category, and each agent is a Person object. Besides the initial state, each agent will be in two states according to their decision strategy, that is, adoption or rejection. In the initial state, a certain proportion of users are selected as seed users in a random way. These users’ status values are initialized as “accept,” and other users’ statuses are initialized as “reject” after the model is started.

The Person category’s parameters and variables are set to realize the evolution of the social network and the statistical data related to the network structure’s characteristics. The main variables and parameters are shown in Table 1.

The detailed descriptions and specific meanings of *b*, *c*, *f*, *m*, *k*, *U_i_*, *U_j_* and other parameters in the model are the same as Zhou et al.’s [7].

### 4.2. The Steps of the Simulation Model

The network evolution mechanism of the existing connection disconnection and reconnection and new connection generation is realized by the connection and disconnection behaviors of each user, and presents the evolution of the social network under the accumulation of user group behaviors. Therefore, we added the steps of connecting and breaking the edge of the user agent (Step 5 and Step 6) in the simulation model. With the methods of “Connectto” and “Disconnectto” provided in the Anylogic 8 Simulation Software, the operation of connecting and breaking edges of each user agent was realized. The breaking and connecting edge are triggered in a probabilistic way. In updating and synchronizing the model data (Step 7), the calculations of the statistical indicators of the social network structure and the statistics of the relevant data were added. It needs to be emphasized that the user’s decision-making and interaction process have not changed (i.e., Step 3 and Step 4). Due to the changes in the social network structure, the objects of interaction and communication with user agents and the network effect value of mobile applications have changed, ultimately impacting the user’s decision-making. The steps of the multi-agent-based simulation model are as follows:

Step 1: Create a certain number of user agents, initialize the agent status, take some users as the initial adopters, and complete the initialization settings of individual preferences, personality characteristics, and other user agents’ parameters.

Step 2: According to the preset network topology type, call the corresponding network generation function to build the network connection between user agents and complete the network structure’s initialization.

Step 3: Operation model. In each operation cycle or time step, the user agent calculates the utility value of the current decision-making mobile application, collects the related parameter information of the neighbor user decision-making, and completes the information collection phase in the user decision-making and interaction process.

Step 4: According to the current decision-making state and the value utility of the mobile application, the user agent adopts a method of intelligent decision-making learning and carries out the decision of approving or rejecting the mobile application in the next step to complete the work of the evolutionary learning stage in the process of user decision-making and interaction.

Step 5: The user agent collects and calculates the information related to the communication and interaction with other user agents, such as the number of time steps that have not occurred learning imitation with the neighbor user agent, the same amount of time steps as other user agents in decision-making, and so forth.

Step 6: According to the new connection generation rule and the existing connection disconnection and reconnection rule, the user agent judges whether the conditions for establishing or disconnecting the connection with other user agents are met. If the conditions are met, it will trigger a probabilistic way to create and disconnect the connection. If the conditions are not met, they will not operate.

Step 7: The model generates statistics on the number of users who currently choose to adopt or reject mobile applications, calculates the statistical indicators of social networks (network average, network aggregation coefficient, network-level path length), generates statistics on the distribution of current network node degrees, and displays them in relevant charts to complete the data update and synchronization of the model.

Step 8: Judge whether the end time of simulation is reached, that is, the preset number of simulation experiment steps. If not, repeat Steps 3 to Step 7; otherwise, proceed to the next step.

Step 9: The model stops running, and the simulation process ends.

**Figure 3:**
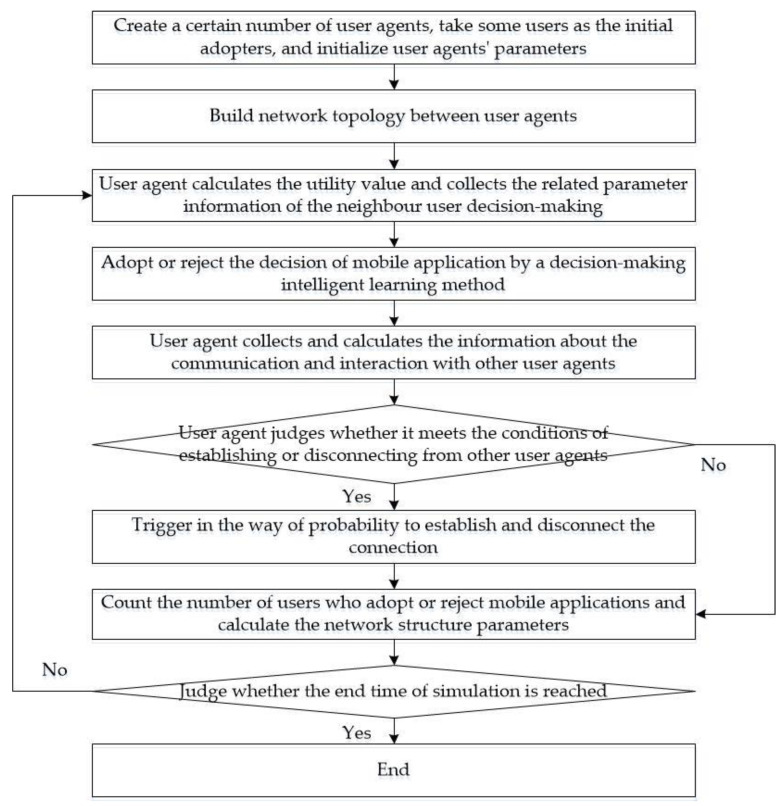
The flowchart of the simulation.

The flowchart of the simulation is shown in Figure 3.

### 4.3. Model Parameter Setting and Verification

The parameters’ settings have a very important impact on the accuracy of the simulation results. Thus, the parameters’ settings of our work referred to Zhou’s [7] and Laciana’s studies [40]. The number of initial users was *n* = 100, and 10% of initial users were seed users. The parameters of mobile application diffusion were set as *b* = 90, *c* = 80, *f* = 55, and *m* = 0. The network effect intensity *d* was set to 10, the user’s individual preference was *Pre* ~ *N* (50, 10), and the user’s characteristic distribution was *ϕ_Cht_* = (0.3,0.3,0.4). The period of each experiment was 100 time steps (days).

**Figure 4:**
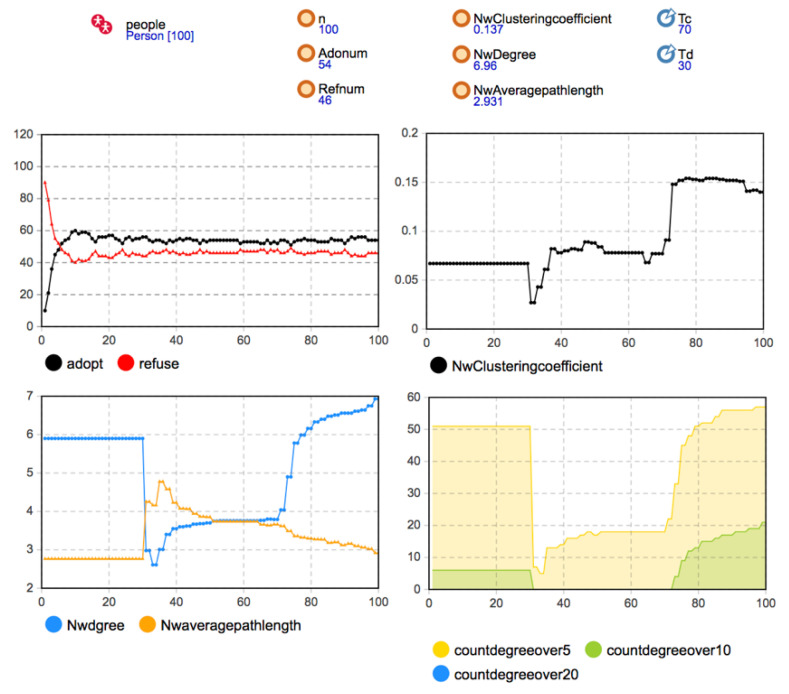
The simulation results by the initial network structure (*b* = 90, *c* = 80, *f* = 55, *m* = 0).

The rules of network evolution were introduced in the process of model construction. Therefore, the validation of the developed model focused on the change of network structure under social network evolution rules. Considering the local network effect, the initial network structure adopted a random network, and the parameters were set as *Td* = 30, *Tc* = 70, *Disconnect* = 0.8, *Reconnect* = 0.3, *Newconnect* = 0.08. The simulation results are shown in Figure 4.

The simulation results show that when *Td* = 30, the aggregation coefficient, the average degree, and the average path length of the social network all had sudden changes. According to the existing disconnection rules of the network, when there is no one-way or two-way decision imitation between neighbor agents in a certain time step, the connection will be disconnected by way of probability triggering. Since the critical time of disconnection was 30, at the 31st time step and later, the agent that reaches the disconnection condition performed the disconnection operation with the probability of *Disconnect* = 0.8. Because of the high triggering probability, the network structure suddenly changed. This is reflected in the network’s statistical indicators. The disconnection of a large number of edges in the network significantly reduced the average degree of the network, the dense connections within the network were reduced to a certain extent, and the aggregation coefficient was significantly reduced. Moreover, because many edges were reduced, the connection between any two nodes required a longer path, so the average path length increased significantly. After *T* = 30, according to the rule of disconnection and reconnection, when the user’s own decision was the same as that of his previous neighbors, the agent’s irregular edge reconnected with the probability of *Reconnect* = 0.3. Therefore, with the connection’s gradual recovery, the aggregation coefficient and average degree of the network gradually increased. The average path length gradually decreased and tended to be stable after about 20 time steps. When *Tc* = 70, the network structure changed again. According to the new connection generation rule, the user and other users who had no contact before had the same decision-making. The user agents generated new connections with the probability of *Newconnect* = 0.08. Hence, the average degree of the network increased again, and the average path length decreased again. It can be seen that the simulation results present the preset network evolution rules, reflect the changes in the network structure in the process of innovation diffusion, and thus, prove the effectiveness of the developed simulation model.

## 5. Simulation Results and Analysis

The simulation experiment explores the laws and characteristics of the coevolution of social networks and innovation diffusion and the role of various internal and external factors in the process of collaborative evolution. To highlight the characteristics of social networks at the micro-level, we fully considered the differences between the user’s neighbor network and the possible impact of the interaction structure changes between individuals on the user’s communication interaction and the value utility of mobile applications. Under the local network effect, the simulation experiment was carried out on the joint evolution of the user group decision and the social network structure. Firstly, we explored the law characteristics of the coevolution of social network and innovation diffusion under the difference of the initial social network structure and the two-way interaction between social network evolution and user decision-making evolution. Then, we analyzed the impact of the perceived benefit, the use cost, marketing investment, and seed users on coevolution.

The simulation model initially sets the total number of users *n* as 100, the user’s preference as *Pre* ~ *N* (50, 10), and the characteristic user distribution always adopts *ϕ_Cht_* = (0.3, 0.3, 0.4), and the user average degree is 6. To distinguish the differences between the two network evolution rules, we set *Td* = 30, and *Tc =* 70. The two evolution rules’ occurrences have specific interval times, which are easier to observe, and *Disconnect* = 0.8, *Reconnect* = 0.3, and *Newconnect* = 0.08. The experimental time was 100 days. In the simulation model, the perceived benefit, cost of use, the proportion of seed users, the intensity of network effect, and other parameters are set according to each part of the simulation experiment’s specific needs.

### 5.1. The Influence of Different Initial Social Network Structures on Coevolution

**Figure 5:**
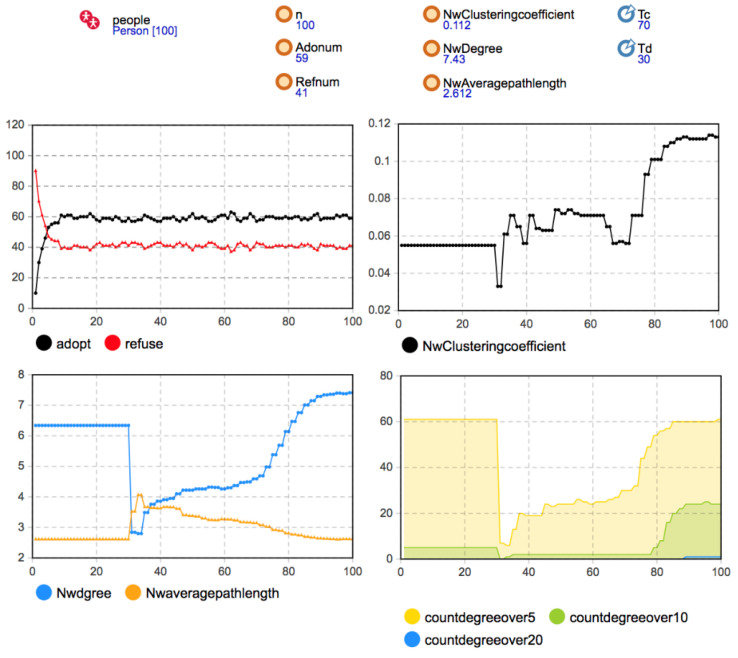
Simulation results when the initial network was s a random network (*b* = 130, *c =* 115, *f =* 55, *m =* 0).

**Figure 6:**
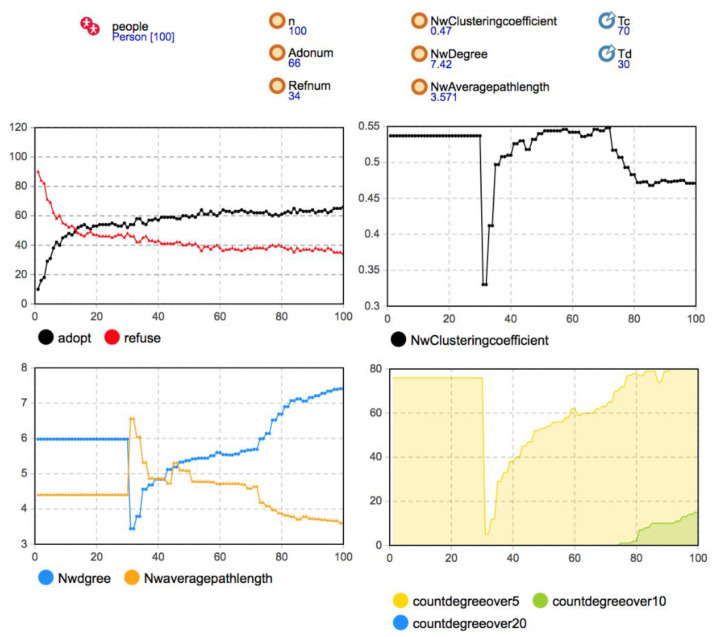
Simulation results when the initial network was a small-world network (*b* = 130, *c =* 115, *f =* 55, *m =* 0).

**Figure 7:**
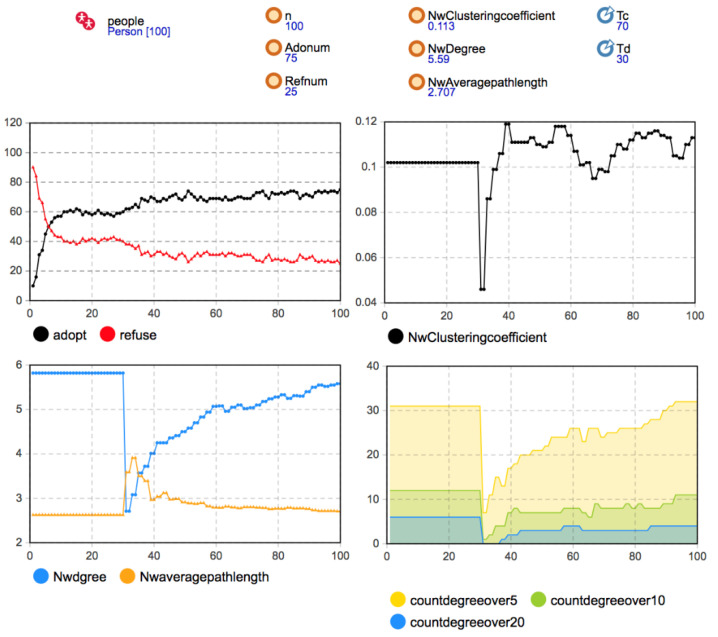
Simulation results when the initial network was a scale-free network (*b* = 130, *c =* 115, *f =* 55, *m =* 0).

The random network, small-world network, and scale-free network were used as the simulation experiment’s initial network structures. The Person category parameters were set as *b* = 130, *c* = 115, *f =* 55, *m =* 0; the network effect intensity *d* was 30; the proportion of seed users was 10%. The simulation results are shown in Figure 5, Figure 6 and Figure 7.

Considering the changes in the network average degree, average path length, and aggregation coefficient, it can be seen that the user agents in the network had no one-way or two-way decision-making imitation in 30 time steps. After the critical time of user disconnection, after *T* = 30, the users began to disconnect with the probability of *Disconnect* = 0.8. At the macro level, many edges in the social network decreased rapidly in a short time, and the aggregation coefficient and average degree of the three network structures were significantly reduced at the original level. The small-world network had an especially higher network aggregation coefficient because of its own small-`world characteristics. A large number of dense connections in the network disappeared due to a large reduction of edges in the network, so the decline of the aggregation coefficient was the most obvious. In terms of average path length, the disconnection of many edges inhibited the three networks’ overall information transmission efficiency, and the average path length increased rapidly. Because the small-world network relies on the long-range edge between some nodes to connect the small groups in the network, the disconnection of some key edges between the nodes in the network led to a longer path between any two nodes. Hence, the average path length of the small-world network increased more obviously. After *T* = 30, due to the reconnection rule of breaking edges, the agents meeting the reconnection condition were reconnected with the probability of *Reconnect* = 0.3. Therefore, with the gradual recovery of the edge in the network, the aggregation coefficient and average degree of the three networks gradually increased, and the average path length gradually decreased. After about 20 time steps, it gradually approached the network’s initial level and became more stable before *T* = 70.

Remark 1: Under the rule of disconnection and reconnection, the reconstruction of the three different network structures was similar in the whole process. The statistical indicators of the network characteristics gradually recovered to the initial level. However, due to the network’s different initial characteristics, the average path length and network aggregation coefficient changes were different.

According to the statistical chart of the number of users adopting and rejecting decisions in Figure 5 and Figure 6, after *T* = 30, the number of adopters in the small-world network and the scale-free network that became stable themselves began to change gradually. With network reconstruction, the diffusion speed accelerated, the mobile application spread further, and the number of adopters gradually increased and reached a relatively stable state after some time. However, when the initial structure was a random network, the user group decision-making was regular with 60 adopters and 40 rejects after *T* = 20. The diffusion degree changed little with time, and the diffusion result was not affected by the network evolution.

Remark 2: The disconnection and reconnection of the existing connections can promote mobile application diffusion in the small-world or scale-free networks, accelerate the diffusion speed in the medium term, and make mobile applications spread further and reach a stable state after some time. Although the random network structure also changed, the user group decision-making was not be affected.

About 30 time steps after the old connection disconnection critical time, the changes in the network average degree and average path length tended to be stable and maintained to the critical time of new connection generation *T* = 70. At this stage, the stability of user group decision evolution was higher than that of other times.

Remark 3: Under the rule of disconnection and reconnection, network structure change urges more users to change their own decisions and adopt mobile applications. Simultaneously, user decision evolution makes the relationship structure between users in social network reconstruct with a higher aggregation, promoting the stability of social network structure and user group decision.

When *T* = 70, according to the new connection generation rule, when the number of time steps for the user agent to make the same decision as other agents without connection reached the critical value *Tc* = 70, the new connection was generated with the probability of *Newconnect* = 0.08.

With the establishment of new connections, the number of adopters in random networks did not change significantly. However, in the small-world network and the scale-free network, the network reconfiguration was similar to that of the one before. Users who chose the same decision-making strategy established new connections, the average path length of the network decreased, and the aggregation coefficient decreased. The evolution of the network structure made the diffusion further deepen. Still, because the change of the network structure was less than the transformation of the network structure in disconnection and reconnection, the influence of user group decision-making is not apparent, and its promotion of diffusion is relatively weak. 

Remark 4: The generation of new connections promotes the diffusion of mobile application innovation in small-world networks and scale-free networks. Compared with the disconnection process and reconnection of existing connections, the new connections’ promotion effect was relatively weak. Meanwhile, in random networks, the evolution of the network structure did not significantly affect user group decision-making.

From the result of coevolution, the scale-free network achieved the best diffusion result. Still, the second was the small-world network. The reason for this is that the social network’s evolution promotes mobile application diffusion, and the number of users who adopt mobile applications increases at different stages. Simultaneously, in user decision evolution, users restore existing connections or generate new connections due to decision consensus, promoting network structure evolution. Meanwhile, when the initial network was a random network, the change in the network structure did not affect the user’s decision-making, so the diffusion result was different from that of the static social network. The diffusion degree of the random network was the lowest.

Remark 5: In the small-world and scale-free networks, social network evolution and user group decision-making evolution promoted each other. The diffusion degree of mobile applications in the user group improved. The best diffusion result was obtained in the scale-free network, followed by the small-world network. The diffusion degree of the random network was the lowest because it was not affected by network evolution.

Considering the change in the network aggregation coefficient, we found that the small-world network’s aggregation degree was stable at the end of the network evolution, slightly lower than the initial level. Compared with the other two network structures, the small-world network’s aggregation coefficient was still significantly higher. Hence, the small-world characteristics still existed. By observing the change in the network degree distribution, it was found that the evolution of social networks had no apparent influence on the number of nodes with a high node degree in the scale-free network. At the final time of diffusion, the number of nodes with a node degree larger than 10 and greater than 20 and their proportion to all nodes did not change significantly. They tended to be stable than the original network, so the heterogeneity of network degree distribution still existed. It was also under the influence of these hub nodes and local network effects that users in the scale-free network were frequently affected by the coevolution process of the social network and innovation diffusion and became adopters. The diffusion speed was faster than the other two networks. Therefore, the diffusion results are consistent with those in the static social networks, and the best diffusion effect under the three network structures was obtained.

Remark 6: In the process of network evolution, the small-world network and the scale-free network can still maintain the network characteristics. Some hub nodes in the scale-free network can continuously promote mobile application diffusion under the local network’s effect, making them have better diffusion speed and impact than the other two network structures.

### 5.2. The Influence of Perceived Benefit and Use Cost on Coevolution

In real life, each person’s identity, social status, and communication scope are different. Therefore, the neighborhood environment between users is different. Meanwhile, there are some opinion leaders with a strong influence. To better reflect this scenario, combined with Remark 6, the degree distribution heterogeneity of the scale-free network was constant in the process of network evolution. Therefore, in the following simulation experiments, the scale-free network was taken as the initial network structure to fit the real scene better and study the impact of mobile applications’ perceived benefit and cost on coevolution.

**Figure 8:**
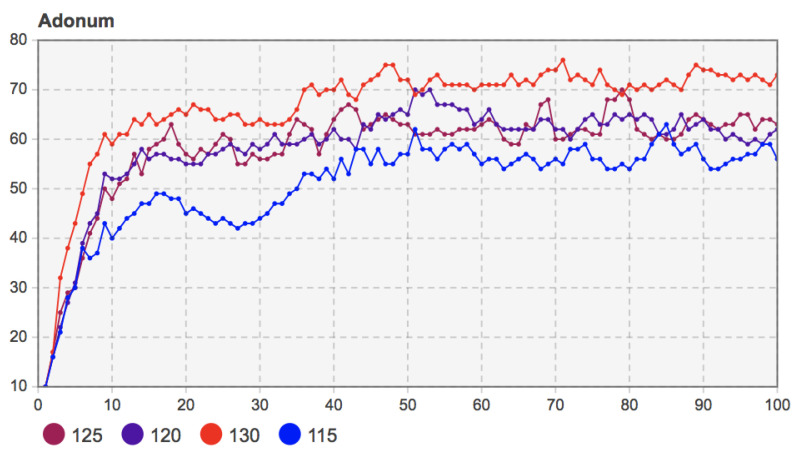
Sensitivity analysis of perceived benefits *b* of mobile applications to the number of adopters (*b* = 115, 120, 125, 130).

(1) The impact of perceived benefit *b* on the coevolution of mobile applications:

The model’s related parameters were set as *c* = 110, *f* = 55, *m =* 20, and the proportion of seed users was 10%. The values of perceived income *b* were taken as 115, 120, 125, and 130, respectively. The simulation results are shown in Figure 8.

Figure 8 shows that when mobile applications’ perceived benefits increase, the number of adopters in the early diffusion stage also increases. Although the trend of group decision-making evolution is similar, mobile applications’ diffusion results with higher perceived benefits are better. For example, when *b* = 130, whether before the network structure changes (*T* = 30) or at the final moment of diffusion, more users chose to adopt. After about 30 time steps of coevolution of social network and innovation diffusion, that is, when *T* = 60, the diffusion under different perceived benefits reached a stable state. At this time, for mobile applications with higher perceived benefits, the degree of user decision-making changes was relatively small, and the change curve of the number of adopters in the final stage of diffusion was relatively stable. This phenomenon is similar to the diffusion scenario of mobile applications and other new products in real life. For mobile applications or products from which users can obtain more value and satisfaction, users can perceive their benefit value, so it is more prudent to choose whether to adopt them or not. For those mobile applications with lower perceived benefits, users are indifferent to it, and their decisions are arbitrary and easily fluctuate.

Remark 7: The increase of the perceived benefit *b* of mobile applications promotes the further diffusion of mobile applications in the user community. This helps the stability of the decision-making evolution of the user community.

**Figure 9:**
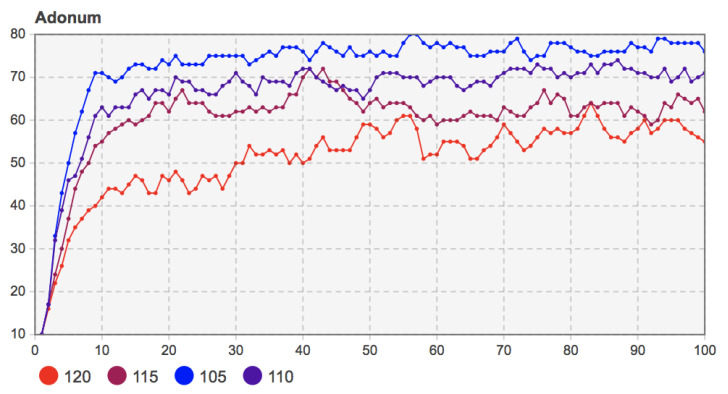
Sensitivity analysis of mobile application use cost *c* to the number of adopters (*c* = 105, 110, 115, 120).

(2) Mobile application use cost *c*:

When the values of the mobile application cost *c* were set as 105, 110, 115, and 120, respectively, the relevant parameters in the model were set as *b* = 130, *f* = 55, *m* = 20, and the proportion of seed users was 10%. The simulation results are shown in Figure 9.

From Figure 9, we can see that with the increase of the use cost, the degree of innovation diffusion of mobile applications was significantly reduced, which means that the use cost affected the user’s willingness to adapt to mobile applications. This effect appeared in the early stage of diffusion. Compared with the use cost *c* = 120, the diffusion speed of the use cost *c* = 105 in the first 10 time steps was significantly faster, and the diffusion effect was better. With the evolution of social networks, the spread of mobile applications in user groups was further deepened. Although the use cost was different, the evolution trend of user group decision-making was still similar. At the final moment, mobile applications with a higher use cost had fewer adopters in the diffusion results. This shows that the differences in mobile applications’ use costs cannot be eliminated through user communication or interactive social learning, or be reflected in the final diffusion results. 

Remark 8: The decrease in use cost *c* increases the diffusion speed in the early stage and increases the final diffusion effect. Moreover, a change in use cost will not affect the trend of user group decision-making evolution. Social network evolution still promotes innovation diffusion, which is similar to the situation of perceived benefit *b*.

### 5.3. The Influence of Marketing Promotion Investment on Coevolution

**Figure 10:**
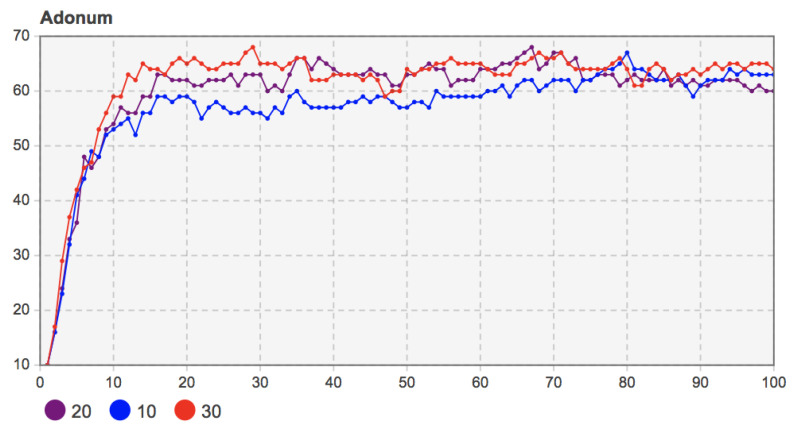
Sensitivity analysis of marketing promotion investment *m* to the number of adopters (*b* = 10, 20, 30).

When the values of marketing promotion investment *m* were 10, 20, and 30, the other relevant parameters were set as *b =* 130, *c* = 120, *f =* 55, and the proportion of seed users was 10%. The simulation results are shown in Figure 10.

Figure 10 shows that the increase of marketing promotion investment *m* increased the users who adopt mobile applications in the early stage of diffusion. However, since *T* = 30, with social networks’ evolution, high marketing promotion investment’s advantages gradually decreased. The mobile applications with less marketing promotion investment gradually expanded, the number of adopters gradually increased, and the promotion effect of marketing promotion investment on diffusion was not obvious. At the end of the diffusion, adopters under the three different marketing investments were much fewer than those in the early diffusion stage. The reason for this is that compared with fewer adopters in the early stage, with the increase of adopters in the later stage under the premise of a specific marketing promotion investment, each adopter gained less value. Under the evolution of social learning behavior and social network, user decision-making was more affected by the network effect and interactive communication. The role of marketing promotion investment weakened. Therefore, the diffusion trend of different marketing promotion investments tended to be the same in the later stage, and the difference of diffusion degree was minimal.

Remark 9: The investment of enterprises in marketing promotion helps promote mobile applications’ users in the new market stage. It has a positive effect on the speed and scale in the early diffusion stage. However, the natural evolution of a social network structure and user group decision-making makes this promotion relatively weak in the later diffusion period. It is difficult to show significant differences in the diffusion results in the later diffusion period.

### 5.4. The Influence of the Number of Seed Users on Coevolution

**Figure 11:**
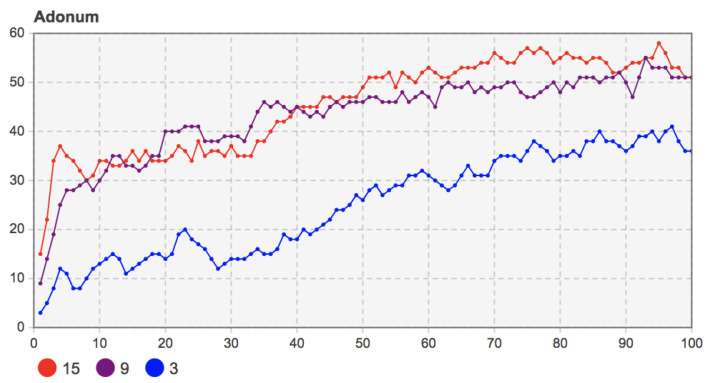
Sensitivity analysis of the proportion of seed users (3%, 9%, 15%) to the number of adopters.

When the proportion of seed users was 3%, 9%, and 15%, the other model parameters were set as *b =* 135, *c =* 130, *f =* 55, *m =* 20. The simulation results are shown in Figure 11.

According to Figure 11, when the proportion of seed users was 9% and 15%, the high proportion of seed users (15%) grew more rapidly under the local network effect. It had more adopters in the initial diffusion period. With the progress of social learning among users and the continuous evolution of social network structure, mobile applications’ diffusion degree in user groups gradually deepened. The number of adopters when the proportion of seed users was 9% increased more quickly than when the proportion of seed users was 15%. At the end of diffusion, the number of users who chose to adopt mobile applications tends to be stable, and the trends under different proportions were similar. The simulation results show that the number of early seed users did not significantly impact the mobile applications’ innovation diffusion. User groups’ social learning behavior and the evolution of the social network structure can make up for early adopter shortages and eventually bring similar diffusion results.

**Figure 12:**
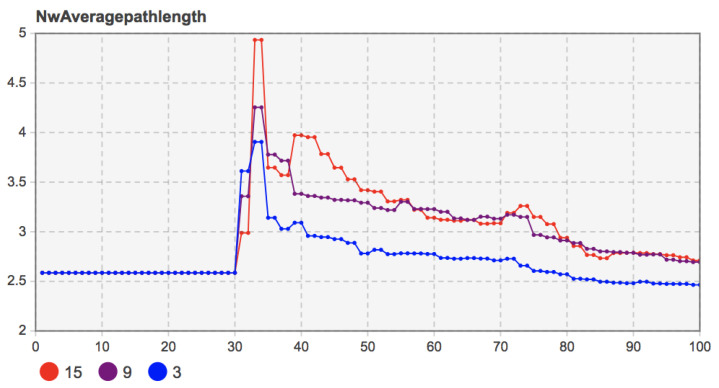
Sensitivity analysis of the proportion of seed users (3%, 9%, 15%) to the network average degree.

**Figure 13:**
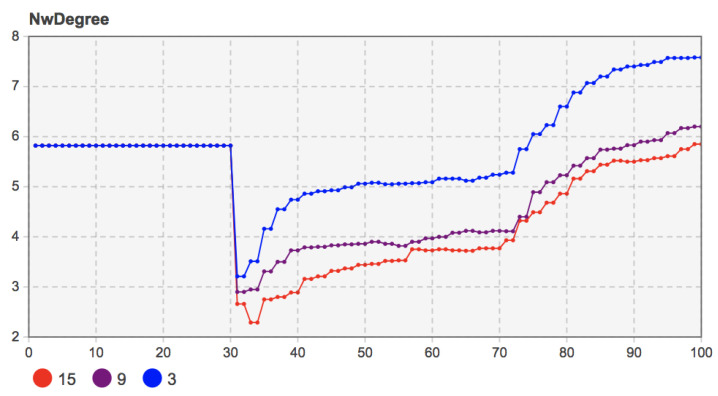
Sensitivity analysis of the proportion of seed users (3%, 9%, 15%) to the average path length of the network.

Further observed were the change in network average degree and the average path length when the proportions of seed users were 3%, 9%, and 15%. The simulation results are shown in Figure 12 and Figure 13. The number of seed users did not affect the overall evolution trend of the social network. In the later stage of innovation diffusion, the average degree and the average path length of the social network with seed users of 9% and 15% were also very similar. That is to say, after the final evolution, the social network structure was similar.

However, in the case of when the proportion of seed users was 3%, due to the relatively low proportion of initial adopters, it was challenging to play the network effect value of mobile applications in the early stage of diffusion under the local network effect. Under the coevolution of the social network structure and user group decision-making, the number of adopters increased gradually. By observing the changes in the average path length, the average path length of the social network in the coevolution stage gradually decreased and was at a low level, which indicates that the system’s information transmission efficiency was high. The number of adopters also presented in the evolution stage of social network structure. A more apparent upward trend was consistent. However, due to the low proportion of initial adopters, the diffusion degree of the final mobile application in the user community could still reach the level under the proportions of seed users at 9% and 15%. The results show that a low proportion of seed users may hinder the diffusion of mobile applications. Only when the number of seed users reached an absolute scale did they not impact the final diffusion results. Otherwise, too few early users also hindered the mobile applications’ diffusion, especially in the early spread of mobile applications. 

Remark 10: The low number of seed users will hinder the spread of mobile apps in the user community. When the number of seed users reaches an absolute scale under the coevolution of social network and user decision-making, the diffusion results and network structure with different seed users tend to be consistent at the final equilibrium time.

## 6. Conclusions

We mainly explored the laws and relationship of coevolution of social networks and innovation diffusion and the role of various internal and external factors in concerted development in order to have more in-depth insight into mobile application diffusion’s internal mechanisms and law characteristics. Firstly, considering the features of social networks’ dynamic changes in real life, we designed the network evolution mechanism of disconnection and reconnection and the new connection generation of social networks for mobile application users. Then, we constructed the multi-agent model of the coevolution of social networks and mobile application diffusion. Under the different conditions of the initial social network structure, the characteristics of the coevolution of the social network and mobile application diffusion and the two-way interaction between social network evolution and user decision-making evolution were explored. Finally, the impact of the value perception benefits, use costs, marketing promotion investments, and the number of seed users on the coevolution of social networks and mobile application diffusion were analyzed. The conclusion is summarized as follows:

(1) Under the rules of network evolution in which existing connections were disconnected and reconnected, and new connections were generated in social networks, social networks’ evolutions processed under different initial network structures were similar in some respects. Due to the different initial characteristics of the network, there were differences in network statistical indicators’ changes.
(2) In the small-world and scale-free network structures, social network structures and user group decision-making evolution promoted each other. The diffusion speed of mobile applications in the medium-term accelerated, and the degree of diffusion in user groups gradually deepened. The scale-free network achieved the best diffusion result, followed by the small-world network. In the random network, the diffusion degree was the lowest because the network evolution did not significantly affect the user group decision-making.
(3) Compared with the generation of new connections in social networks, the disconnection and reconnection of existing connections had a more substantial role in promoting innovation diffusion. Under this rule, network structure evolution urged more users to change their own decisions and adopt mobile applications. Meanwhile, users’ decisions made the relationship structure between users in the social network reconstruct with a higher aggregation, which further promoted social network structure and a user group decision.
(4) Small-world network and scale-free network could still maintain their characteristics in the process of social network evolution. Some hub nodes with higher node degrees existed in the scale-free network under the heterogeneity of degree distribution, which continuously promoted mobile applications’ innovation diffusion under the local network effect to a better diffusion speed and impact than the other two network structures.
(5) The increase of mobile applications’ perceived benefits promoted mobile application diffusion in user groups and contributed to group decision-making stability. Reductions in use costs can increase the diffusion speed in the initial stage and improve the final diffusion effect. Although the perceived benefits and use costs did not affect the promotion of social network evolution on innovation diffusion, they were still the key factors to responding to the value utility of mobile applications to users and fundamentally affected user decision-making. Moreover, the differences in the perceived benefits and the use of mobile applications could not be alleviated through social learning and network evolution. This was reflected in the diffusion results.
(6) The investment of enterprises in marketing and promotion positively impacted mobile applications’ diffusion speed and scale in the early stage. However, social networks’ coevolution and user group decision-making made this promotion relatively weak in the late stage of diffusion. It was challenging to show apparent differences in the diffusion results.
(7) A low number of seed users hindered the spread of mobile applications in the user community. However, when the number of seed users reached an absolute scale, under the coevolution of the social network and user decision-making, the diffusion results and the network structure with different seed users tended to be consistent at the final equilibrium time.

According to the conclusions of mobile application innovation diffusion, we put forward the following suggestions for implementing and developing mobile application innovation diffusion of Internet companies. Firstly, companies should provide comprehensive and practical functions, and good performance and support for mobile applications’ free use. Secondly, they should strengthen the interactions between user circles and attach importance to the operation of the user community. Thirdly, the companies should encourage opinion leaders to become adopters as soon as possible, seize the market opportunities, and further strengthen opinion leaders’ roles in promoting diffusion by building communication bridges with users. Fourthly, in the initial stage of diffusion, marketing promotion investment needs to ensure a certain scale of seed users, but there is no need to blindly pursue the number of seed users.

However, there are some limitations to our work. For example, the conceptual design of the multi-agent model seriously lacks justifications and grounding based on empirical data [41]. In future work, we will collect new empirical data to improve the simulation result’s accuracy.

## Figures and Tables

**Table 1:** The main variables and parameters.

Variables and Parameters	Explanations
*Adonum*	The number of users who choose to adopt the mobile application user’s policy
*Refnum*	The number of users who choose to reject the policy
*n*	The total number of mobile application users
*Td*	The critical time step of connection disconnection between neighboring users
*Tc*	The critical time step of connection generation between neighboring users
*NwDegree*	The average degree of the social network
*NwAveragepathlength*	The average path length of the social network
*NwClusteringcoefficent*	The social network’s aggregation coefficient
*Cht*	The personality characteristics; *Cht* = {1, 2, 3} represents conservative, balanced, and variable types, respectively
*Pre*	The user’s preference for mobile applications; the value follows the normal distribution *N* (*u*, *w*)
*Deatti*	Whether the user rejects the mobile application at the time of *t* (day)
*ra*1 and *ra*2	The probability that the user with the same day’s decision state of reject changes to adopt and the user with the same day’s decision state of adopting changes to reject
*Adnra*	The proportion of the users who choose to adopt the policy among all the neighborhood users
*Idex*	The agent subscript
*Iree*	The degree value, that is, the number of neighborhood users connected to the user
*h*	The number of users in the user’s current neighborhood
*studex*	The agent subscript value of the imitated object when the user’s imitative behavior occurs one day
*staj*	The decision-making state of the decision-making learning object
*chaj*	The personality characteristics of the decision-making learning object
*Discon*	The triggering probability of an agent to agent connection disconnection after meeting the existing connection disconnection condition of network evolution
*Recon*	The triggering probability of an agent to agent connection reconnect after meeting the existing connection reconnect condition
*Newcont*	The triggering probability of an agent to agent new connection generation after meeting the new connection generation condition
*InIree*	The number of neighbor users connected to itself in the first social network
*Cltor*	The aggregation coefficient of the user’s neighbor network
*Diall*	The sum of the shortest path length from the user to all other users
*arrt*	The time steps that have not occurred one-way or two-way imitation with the neighbor user agent
*arridex*	The subscript of the neighbor agent
*arradt*	The same number of time steps as other user agent decisions
*arrde*	The network adjacency matrix of the social network

## Data Availability

Data sharing not applicable.
